# A Case in Which the Endoscopic Denker’s Approach Was Useful in the Diagnosis of IgG4-Related Ophthalmic Disease

**DOI:** 10.7759/cureus.86809

**Published:** 2025-06-26

**Authors:** Yusei Yamaguchi, Shinya Ohira, Ryousuke Yui, Satoshi Sonobe, Kota Wada

**Affiliations:** 1 Otolaryngology, Toho University, Tokyo, JPN; 2 Otolaryngology, The Jikei University School of Medicine, Tokyo, JPN; 3 Pathology, Toho University, Tokyo, JPN

**Keywords:** endoscopic sinus surgery, optic nerve compression, orbital tumor, probable, steroids

## Abstract

IgG4-related disease (IgG4-RD) is a chronic inflammatory condition characterized by elevated serum IgG4 levels, infiltration of IgG4-positive plasma cells, and fibrosis in various organs. We report the case of a 76-year-old man who presented with left-sided proptosis. Computed tomography revealed a mass lesion in the left orbit. An initial biopsy via a transnasal approach under local anesthesia was inconclusive. Although endoscopic sinus surgery was performed under general anesthesia, a definitive diagnosis could not be obtained. The lesion continued to enlarge, and subsequent ophthalmologic examinations revealed progressive optic nerve compression. Therefore, tumor resection was performed again under general anesthesia using the endoscopic Denker’s approach. The tumor was successfully resected without complications. Histopathological findings led to a diagnosis of probable IgG4-related ophthalmic disease (IgG4-ROD). Following surgery, the residual lesion enlarged again; however, a three-day course of steroid pulse therapy resulted in reduction of the lesion and improvement of optic nerve compression. The patient has remained relapse-free. While 81% of IgG4-ROD cases involve the lacrimal gland, other orbital structures such as the pterygopalatine fossa, trigeminal nerve branches, extraocular muscles, orbital fat, eyelids, and nasolacrimal duct can also be affected. In cases without lacrimal gland involvement, the optimal approach for obtaining diagnostic biopsy specimens should be considered individually. Although there is no consensus on the required volume of tissue for diagnosis, we believe that aggressive resection of the central lesion is necessary for accurate diagnosis. The endoscopic Denker’s approach facilitates wide exposure and resection of far lateral maxillary sinus lesions, enabling both decompression and definitive diagnosis, which can lead to appropriate subsequent treatment.

## Introduction

Immunoglobulin G4-related disease (IgG4-RD) is a chronic inflammatory disorder characterized by elevated serum IgG4 levels, infiltration of IgG4-positive plasma cells, and fibrosis in various organs [1]. The disease can affect multiple organs, including the lacrimal and salivary glands, lungs, pancreas, gallbladder, kidneys, and retroperitoneum [2]. Among Asian populations, involvement of the head and neck region, particularly the lacrimal and salivary glands, has been reported more frequently [3]. Regardless of the affected organ, corticosteroids are generally considered the first-line treatment [4].

In this report, we present a case in which the endoscopic Denker’s approach was useful for the diagnosis of IgG4-related ophthalmic disease (IgG4-ROD), along with a brief review of the relevant literature.

## Case presentation

A 76-year-old man (weight: 64 kg) presented with progressive left-sided proptosis. His medical history included hypertension and non-insulin-dependent diabetes mellitus. He had undergone endoscopic sinus surgery (ESS) at another hospital 20 years prior, although the original indication was unclear. The patient had noticed left-sided proptosis for over a decade but did not seek medical attention due to the absence of pain or visual symptoms. Following a traffic accident, a head CT performed at another hospital incidentally revealed a mass lesion in the left orbit, and he was referred to our department and the ophthalmology department for further evaluation.

**Figure 1:**
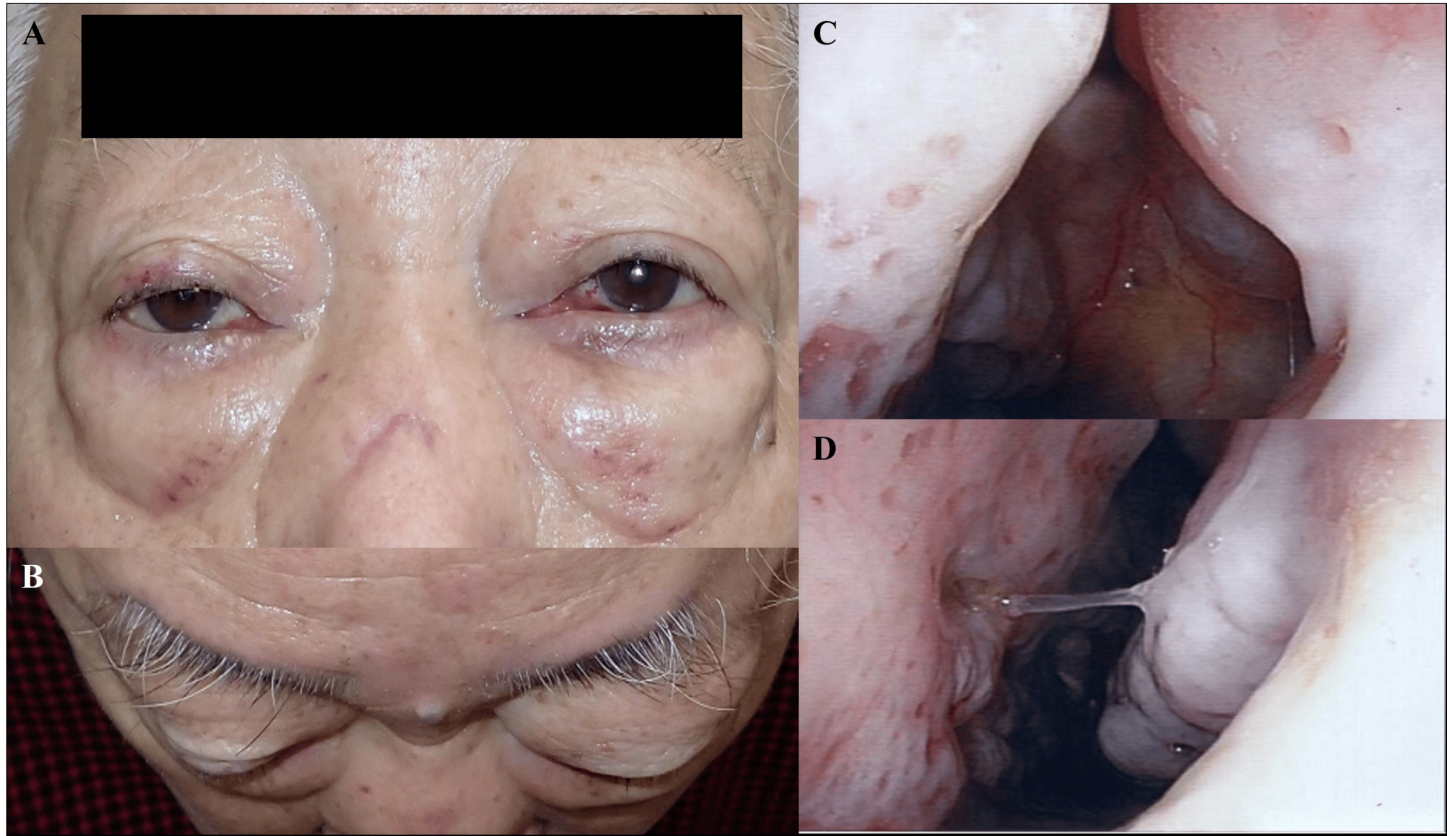
Clinical Photographs and Nasal Endoscopic Images A: Frontal view showing mild left proptosis. B: Superior view also demonstrating mild left proptosis. C: Nasal endoscopy of the left superior nasal cavity showing the absence of the superior and middle turbinates. D: Endoscopic view of the left inferior nasal cavity, where the inferior turbinate appears slightly atrophic.

On initial examination, left-sided proptosis and restricted upward and lateral movement of the left eye were noted (Figure 1A, 1B). There was no diplopia, visual acuity loss, or visual field deficit. Nasal endoscopy showed the absence of the superior and middle turbinates, but no obvious intranasal tumor (Figure 1C, 1D).

**Figure 2:**
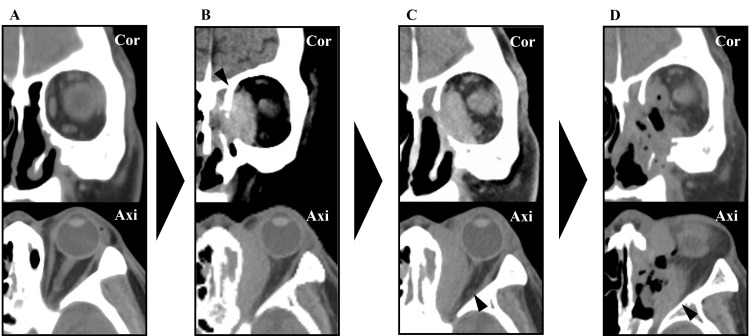
Paranasal Sinus CT Images A: CT scan taken 15 years prior showing an intact medial orbital wall with no evidence of orbital mass; medial rectus and optic nerve appear normal. B: Initial CT at presentation revealing a 45 × 18 mm mass (arrowhead) along the medial wall of the left orbit, invading into the sinus and showing unclear margins with the medial rectus and optic nerve. C: Coronal CT eight months after first surgery showing tumor progression, with invasion into the medial rectus, superior oblique, and inferior rectus muscles; the mass occupies half of the orbit and compresses the optic nerve (arrowhead). D: Postoperative CT after second surgery showing substantial tumor removal; however, residual tumor remains in the anterior and lateral orbit (arrowhead).

**Figure 3:**
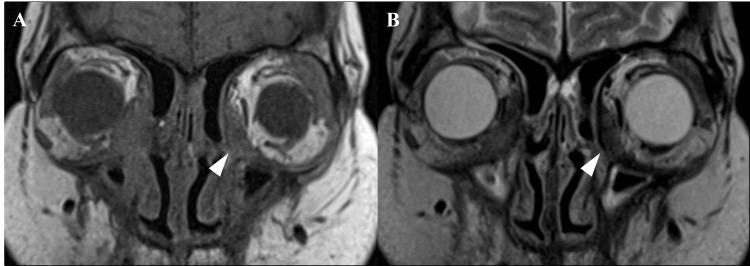
Paranasal Sinus MRI Images A: T1-weighted image showing isointense orbital mass (arrowhead) appearing to invade the medial rectus. B: T2-weighted image showing the mass as hypointense (arrowhead).

Blood test results showed that the total IgG level was 1,340 mg/dL (reference range: 861-1,747 mg/dL) and the IgG4 level was 76.7 mg/dL (reference range: 11-121 mg/dL), both within normal limits, while tumor markers and antineutrophil cytoplasmic antibodies (ANCA) were within normal ranges. Sinus CT demonstrated a 45 × 18 mm slightly hyperattenuating mass along the medial wall of the left orbit, with bony destruction (Figure 2B). This lesion was not present on a CT scan taken 15 years earlier (Figure 2A). MRI showed the mass to be hyperintense on T1-weighted and hypointense on T2-weighted images (Figure 3A, 3B). An outpatient biopsy via a transnasal approach targeting the medial orbital wall was non-diagnostic. Suspecting malignancy or an inflammatory pseudotumor, we proceeded with endoscopic sinus surgery (ESS) under general anesthesia for diagnostic purposes.

First surgery

**Figure 4:**
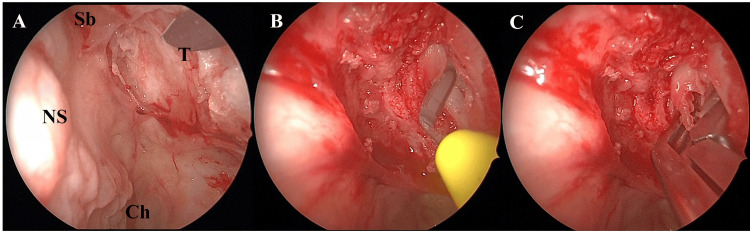
Intraoperative Views from the First Endoscopic Sinus Surgery A: Observation of the medial orbital wall from the nasal cavity; nasal mucosa was removed to expose the tumor. NS: Nasal septum; Sb: Skull base; Ch: Choana; T: Tumor. B: Tumor incised with a crescent knife; no bleeding observed. C: Part of the orbital tumor was removed with forceps; the mass was white and firm.

One month after the initial consultation, ESS was performed under general anesthesia (Figure 4). The uncinate process and both the middle and superior turbinates were absent. Using a navigation system, we identified the medial orbital wall and incised the mucosa with a sickle knife (Figure 4A, 4B). Part of the tumor was excised with upward-curette forceps (Figure 4C), and the frontal sinus drainage pathway was opened before concluding the procedure. No postoperative complications occurred, and the patient was discharged on postoperative day three. Histopathology showed only low-cellularity fibrous tissue, and a definitive diagnosis could not be made. Given the absence of malignant features, the patient was followed regularly with imaging and ophthalmologic exams.

Progression

Eight months after the initial surgery, the tumor gradually enlarged, and signs of optic nerve compression appeared on ophthalmologic exams (Figure 2C). Concerned about potential vision loss, we opted for reoperation to both relieve the symptoms and obtain a more extensive specimen for diagnosis.

Second surgery

**Figure 5:**
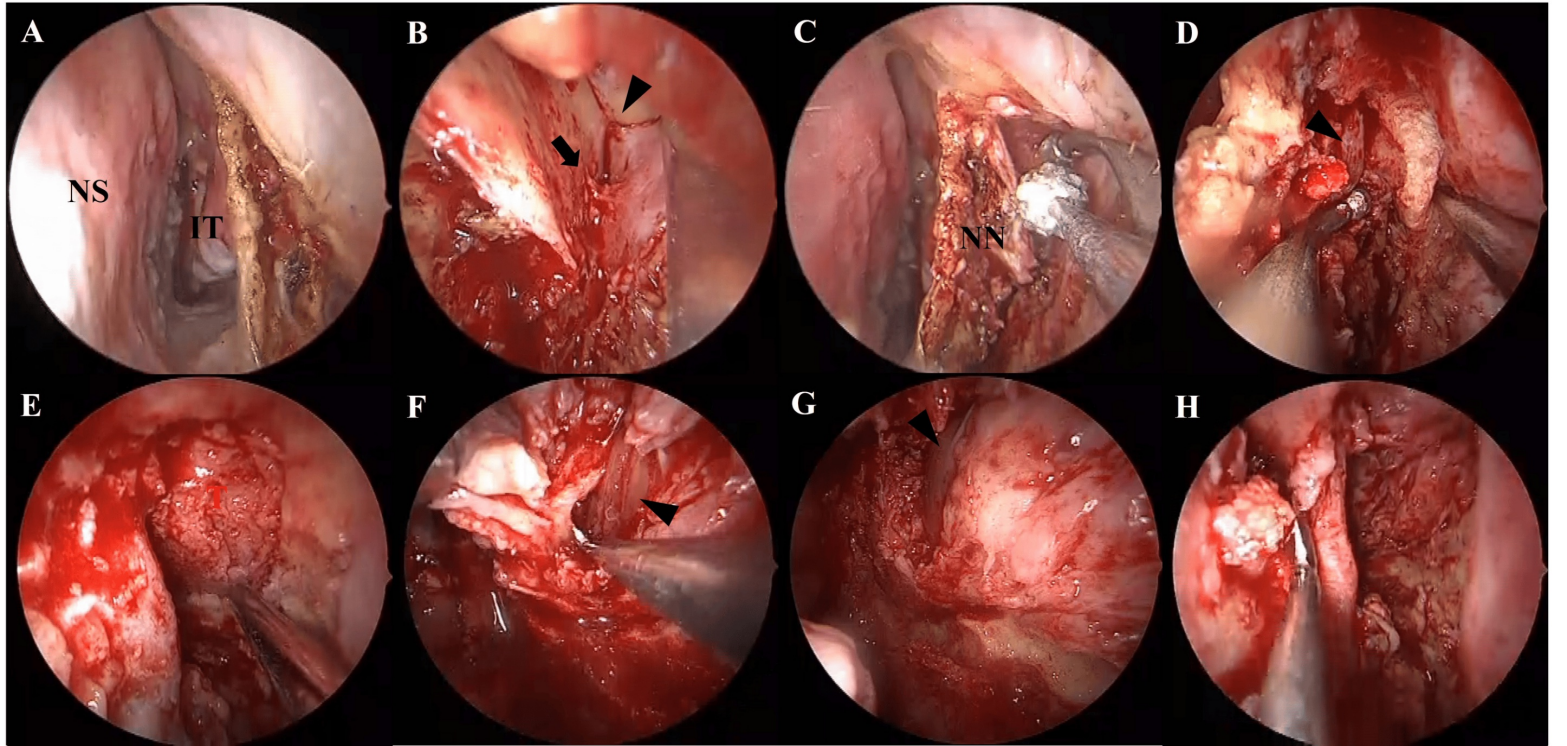
Intraoperative Views from the Second Surgery Using the Endoscopic Denker's Approach NS: Nasal septum; IT: Inferior turbinate; T: Tumor; NN: Nasal notch. A: Incision made anterior to the inferior turbinate at the level of the piriform aperture. B: Infraorbital nerve (arrowhead) and artery (arrow) identified; artery was cauterized and divided. C: Soft tissue around the nasal notch was dissected, and bone was drilled. D: Nasolacrimal duct (arrowhead) was displaced medially to expose the orbital floor. E: Orbital floor and remaining medial wall were drilled to widely expose the orbital tumor. F: Tumor was removed in segments, separated from the inferior rectus muscle (arrowhead). G: Tumor debulked, revealing the medial rectus muscle (arrowhead). H: Bone removal extended near the optic nerve; tumor was maximally resected, and the surgery was completed.

Fourteen months after the first surgery, we performed a second ESS under general anesthesia using the endoscopic Denker’s approach. An incision was made anterior to the inferior turbinate at the level of the piriform aperture (Figure 5A). The infraorbital nerve (arrowhead) and artery (arrow) were identified, and the artery was cauterized and divided (Figure 5B). Surrounding soft tissues were dissected and bone was removed to expose the orbital floor (Figure 5C). The medial and inferior orbital walls were drilled open to widely expose the tumor (Figure 5D, 5E). The mass was removed in multiple pieces. The inferior and medial rectus muscles were visualized (Figure 5F, 5G). Bone removal was extended near the optic canal, and near-total resection of the tumor was achieved. Hemostatic packing material was placed in the ethmoid sinus before concluding the procedure (Figure 5H).

Postoperative course

**Figure 6:**
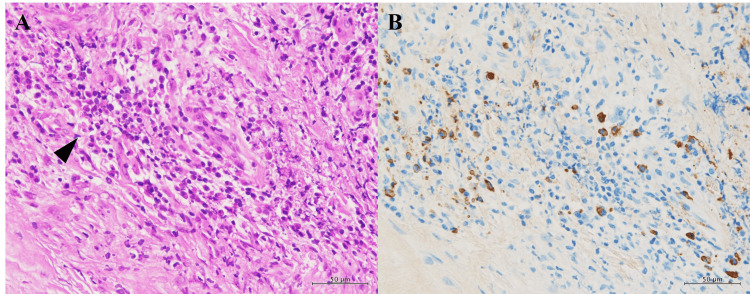
Histopathological Findings of the Excised Orbital Tumor A: Dense fibrous connective tissue with focal aggregation of plasma cells (arrowhead). B: IgG4-positive plasma cells accounted for more than 50% of IgG-positive cells, with up to 25 cells per high-power field (HPF).

The patient again experienced no complications and was discharged on postoperative day three. The proptosis and optic nerve compression improved markedly (Figure 2D). Histopathological examination revealed that more than half of the IgG-positive plasma cells were IgG4-positive, with peak counts of approximately 25 cells per high-power field (HPF) (Figure 6A, 6B).

Based on the imaging and histological findings, the diagnosis of probable IgG4-ROD was made. Whole-body CT showed no evidence of other IgG4-related organ involvement. Two months after the second surgery, the residual lesion again enlarged, and optic nerve compression recurred. A three-day course of steroid pulse therapy (methylprednisolone sodium succinate 500 mg/day) was administered, resulting in tumor shrinkage and symptom relief. To prevent recurrence, oral prednisolone was initiated at 30 mg/day (0.5 mg/kg), tapered monthly, and currently maintained at 10 mg/day. As of 18 months after the second surgery, the patient remains under joint follow-up with otolaryngology, ophthalmology, and rheumatology without evidence of recurrence.

## Discussion

IgG4-RD is a chronic inflammatory condition characterized by elevated serum IgG4 levels, infiltration of IgG4-positive plasma cells, and fibrosis in various organs [1]. Commonly affected sites include the lacrimal glands, salivary glands, lungs, pancreas, gallbladder, kidneys, and retroperitoneum [2]. In Asian populations, lesions are more frequently observed in the head and neck region, particularly in the lacrimal and salivary glands [3].

With accumulating case reports, organ-specific diagnostic criteria for IgG4-RD have been developed. In 2014, diagnostic criteria for IgG4-ROD were established [5], comprising: (1) radiologic evidence of hypertrophic lesions in orbital structures such as the lacrimal gland, trigeminal nerve, and extraocular muscles; (2) histopathological findings of dense lymphoplasmacytic infiltration, fibrosis, and IgG4-positive plasma cells, with an IgG4/IgG-positive cell ratio >40% or >50 IgG4+ cells per HPF; and (3) elevated serum IgG4 (>135 mg/dL). A definitive diagnosis (definite) requires all three criteria; probable diagnosis requires (1) and (2); and possible diagnosis requires (1) and (3) [5]. Revised criteria in 2023 emphasized optic neuropathy as the most severe manifestation of IgG4-ROD [6]. However, 30% of IgG4-RD patients have normal serum IgG4 levels despite characteristic clinical and pathological features [7]. Due to non-specific radiologic findings, differentiating IgG4-RD from other diseases is often challenging [8].

Although 81% of IgG4-ROD cases involve the lacrimal glands, other sites such as the pterygopalatine fossa, trigeminal branches, extraocular muscles, orbital fat, eyelids, and nasolacrimal duct may also be involved [9]. In our case, the lesion was limited to the orbital fat. Lacrimal gland lesions are often bilateral, and many cases previously diagnosed as Mikulicz’s disease, idiopathic orbital inflammation, orbital pseudotumor, or pseudolymphoma may in fact represent IgG4-ROD [10]. IgG4-ROD commonly presents with chronic, painless orbital swelling and proptosis, sometimes accompanied by peripheral lymphadenopathy [11]. Visual acuity is usually preserved, but vision loss due to optic nerve compression has been reported [12], as was possible in our case without timely reoperation.

At the time of the second surgery, the diagnosis of IgG4-ROD had not yet been established, leaving surgical resection as the only option. As noted above, differentiating IgG4-RD from other diseases solely based on imaging findings remains highly challenging [13]. Nonetheless MRI is reported to be helpful in the diagnosis, with T1-weighted images showing iso- to high-intensity and T2-weighted images showing low intensity [14], consistent with our findings. Because lesions frequently involve the lacrimal glands, a pathological diagnosis is often established by biopsy of lacrimal gland tissue, as reported in numerous studies [15-20]. On the other hand, the optimal biopsy approach for lesions located exclusively in other orbital tissues must be considered on a case-by-case basis. Our literature review identified 13 biopsy-confirmed cases of non-lacrimal IgG4-ROD [21-28]: 10 via anterior orbital approaches [21-26], two via orbital exenteration [26,27], one transcranial approach [28], and our own case via a transnasal route.

Of these, three cases (including ours) required re-biopsy due to initial diagnostic failure, often because the specimen volume was insufficient. Our initial two biopsies were limited in volume and taken from the lesion periphery, likely contributing to the delayed diagnosis. While no standard biopsy volume has been established, we believe that large-volume sampling from the lesion's core is essential. In our case, the tumor extended subcutaneously, and an anterior orbital approach might have been considered. Collaborative planning with ophthalmology, dermatology, plastic surgery, and neurosurgery is necessary to determine the most appropriate surgical approach. Steroid therapy is the mainstay treatment for IgG4-ROD, typically resulting in rapid improvement of mass size, symptoms, and serum IgG4 levels within weeks [29]. However, relapse after steroid cessation is common, and maintenance therapy with low-dose prednisolone (5-10 mg/day) or combination with immunosuppressants is recommended [30]. Notably, probable IgG4-ROD with normal serum IgG4 is considered a risk factor for poor steroid responsiveness [31], necessitating continued follow-up as in our case. Complementary treatments include methotrexate, azathioprine, mizoribine, mycophenolate mofetil, anti-TNF-α agents (infliximab, adalimumab), rituximab, radiation therapy, and surgical resection [32].

Surgical reduction may reduce the need for postoperative steroid use and decrease recurrence risk. Iwasaki et al. reported that larger resections resulted in reduced steroid dependence [33], and Ominato et al. reported a 13.3% recurrence rate after 70-100% lesion resection, significantly lower than the 30-70% reported after steroid therapy alone [34]. While the risk of damaging extraocular muscles or the optic nerve must be considered, aggressive resection is often critical for both diagnosis and relapse prevention.

Considering surgical procedure, the endoscopic Denker’s approach expands the piriform aperture by removing the anterior maxillary bone, enabling wide exposure of the anterior, inferior, and lateral maxillary sinus walls, and access to the pterygopalatine and infratemporal fossae [35,36]. It facilitates resection of lesions in far-lateral regions of the maxillary sinus not reachable with standard ESS [37]. Potential complications include injury to the infraorbital nerve, pterygoid plate collapse, and nasolacrimal duct damage [37]. In our case, the left maxillary sinus was underdeveloped post-ESS, precluding access via endoscopic modified medial maxillectomy (EMMM) or the Caldwell-Luc approach. The endoscopic Denker’s approach provided direct visualization of the orbital mass, allowing successful resection despite its risks.

Early biopsy or surgery is essential when orbital masses are identified, even if serum IgG4 is normal. IgG4-ROD should remain in the differential diagnosis, and multiple biopsies or surgeries may be necessary. In our case, cooperation with ophthalmology enabled prompt detection of optic nerve compression. In anatomically normal patients, EMMM or Caldwell-Luc may offer less invasive access, but in our surgically altered anatomy, the Denker’s approach was optimal. Despite its risks, the endoscopic Denker’s approach allowed wide tumor resection, facilitating diagnosis.

## Conclusions

We report a case in which repeated transnasal biopsies failed to yield a diagnosis, but the endoscopic Denker’s approach enabled wide resection of an orbital mass, leading to a diagnosis of IgG4-ROD. Although IgG4-RD is characterized by elevated serum IgG4 and infiltration of IgG4-positive plasma cells, 30% of patients may have normal IgG4 levels. Additionally, non-specific imaging features complicate differential diagnosis. When orbital tumors are encountered, IgG4-ROD should be considered even if serum IgG4 is normal. Multiple biopsies or surgeries may be necessary for diagnosis. In our patient, previous sinus surgery had obliterated the maxillary sinus, making standard approaches such as EMMM or Caldwell-Luc infeasible. The endoscopic Denker’s approach allowed successful resection and definitive diagnosis. For cases where limited biopsy fails to confirm the diagnosis, collaborative evaluation of alternative approaches, including the endoscopic Denker’s approach, is warranted for effective diagnosis of orbital IgG4-ROD.
